# Supplementary material for: Discovery of exolytic heparinases and their catalytic mechanism and potential application
Source: Nat Commun. 2021 Feb 24;12:1263. doi: 10.1038/s41467-021-21441-8 (PMC7904915; doi:10.1038/s41467-021-21441-8)
Supplement: Supplementary file 1 — Supplementary Information [file 41467_2021_21441_MOESM1_ESM.pdf]

Supplementary materials for this manuscript include the following:

Supplementary Information for

Discovery of exolytic heparinases and their catalytic mechanism and potential application

Qingdong Zhang, Hai-Yan Cao, Lin Wei, Danrong Lu, Min Du, Min Yuan, Deling Shi, Xiangxue Chen, Peng Wang, Xiu-Lan Chen, Lianli Chi, Yu-Zhong Zhang and Fuchuan Li

Corresponding authors: fuchuanli@sdu.edu.cn and zhangyz@sdu.edu.cn

Supplementary Information Text

Supplementary Methods

Preparation and Sequencing of Structure-defined HP Tetrasaccharides. Size-defined HP tetrasaccharide fraction prepared from the partial product of HP by Hepase II was further fractionated by anion-exchange HPLC on a Pack Polyamine II column eluted using a NaH_2PO_4 gradient (0.15-1 M) by monitoring at 232 nm. The main peaks (P4-1, P4-2, P4-3, P4-4, P4-5, P4-6, P4-7 and P4-8) were collected and desalted through size exclusion chromatography (SEC) on a Superdex Peptide 10/300 GL column. Then the fractions were freeze-dried repeatedly to remove NH_4HCO_3 to get the purified tetrasaccharide subfractions.

To determine the disaccharides composition of the tetrasaccharide subfractions (P4-4, P4-5, P4-6, P4-7 and P4-8), 5 pmol of each sample was exhaustively degraded by a mixture of Hepase I, II and III, respectively. Then the products were 2-AB labeled and analyzed by anion-exchange HPLC on a Pack Polyamine II column eluted using a NaH_2PO_4 gradient (0-1 M) and monitored using a fluorescence detector with excitation and emission wavelengths of 330 and 420 nm, respectively.

To determine the reducing end disaccharides of the tetrasaccharide subfractions (P4-4, P4-5, P4-6, P4-7 and P4-8), 5 pmol of each tetrasaccharide were primarily treated with 2-*O*-sulfatase[1] and glucuronidase[2] or O_3[3] followed by exhaustively degraded by Hepases (I, II and III). Then the products were 2-AB labeled and analyzed by anion-exchange HPLC on a Pack Polyamine II column as described above.

To determine the type of uronic acids in the reducing end disaccharides of the tetrasacchride subfractions, 400 μg of each tetrasaccharide subfraction (P4-4, P4-5, P4-6, P4-7 or P4-8) were analyzed using ^{1}H NMR spectroscopy, respectively. The type of the

internal uronic acids in each tetrasaccharide preparation were directly determined by ^{1}H NMR spectroscopy as described previously[4]. ^{1}H NMR spectroscopy was performed on a JNM-ECP600 (JEOL, Japan) instrument set at 600 MHz. Each sample (400 μg) was dissolved in 0.5 ml of D_2O in a 5 mm NMR tube.

Supplementary Results

Preparation of Structure-defined HP Tetrasaccharide Subfractions. To prepare a series of structure-defined tetrasaccharides, the size-defined tetrasaccharide fraction prepared from the partial digestion of HP was further fractionated on anion-exchange HPLC as described above. As a result, eight main fractions, P4-1, P4-2, P4-3, P4-4, P4-5, P4-6, P4-7 and P4-8, were obtained (Supplementary Fig. 6). To determine the disaccharide composition of the five main fractions (P4-4, P4-5, P4-6, P4-7 and P4-8), they were exhaustively degraded by Hepases I, II and III, followed by 2-AB labeling and analysis by anion-exchange HPLC. The results in Supplementary Fig. 7a-f show the disaccharide composition of each tetrasaccharide subfractions as follows: P4-4 contained two disaccharides ΔUA(1–4)GlcNAc6S and ΔUA(1–4)GlcNS6S with a 1:1 molar ratio; P4-5 contained ΔUA(1–4)GlcNS and ΔUA(1–4)GlcNS6S with a 1:1 molar ratio; P4-6 contained ΔUA(1–4)GlcNS6S only; P4-7 contained ΔUA(1–4)GlcNS6S and ΔUA2S(1–4)GlcNS6S with a 1:1 molar ratio; and P4-8 contained ΔUA2S(1–4)GlcNS6S only.

To determine the reducing end disaccharide of the tetrasaccharides, fractions P4-4, P4-5, P4-6, P4-7 and P4-8 were initially treated with 2-*O*-sulfatase and glucuronidase or O_3 to remove the unsaturated nonreducing end uronic acid and then exhaustively degraded by Hepases I, II and III followed by 2-AB labeling and analysis by

anion-exchange HPLC as described above. Due to the destruction of the unsaturated disaccharides at the nonreducing ends, the reducing end disaccharides of these tetrasaccharides could be easily determined by HPLC. As shown in Supplementary Fig. 7a-f, the reducing end disaccharides of P4-4, P4-5, P4-6, P4-7 and P4-8 are ΔUA(1–4)GlcNS6S, ΔUA(1–4)GlcNS6S, ΔUA(1–4)GlcNS6S, ΔUA2S(1–4)GlcNS6S and ΔUA2S(1–4)GlcNS6S, respectively.

To determine the uronic acid type of the reducing end disaccharides of P4-4, P4-5, P4-6, P4-7 and P4-8, these tetrasaccharide samples were individually analyzed by ^{1}H NMR spectroscopy. As shown in Supplementary Fig. 8, the types of internal uronic acids in tetrasaccharide subfractions P4-4, P4-5, P4-6, P4-7 and P4-8 were IdoA/GlcA, IdoA/GlcA, GlcA, GlcA and IdoA2S, respectively.

Taken together, based on the disaccharide compositions, the reducing end disaccharides and the internal uronic acid types of the tetrasaccharides, the sequences of P4-4, P4-5, P4-6, P4-7 and P4-8 can be determined as shown in Supplementary Table 1. The P4-4 and P4-5 fractions are mixtures of two isomers containing IdoA and GlcA, respectively.

Supplementary Figures

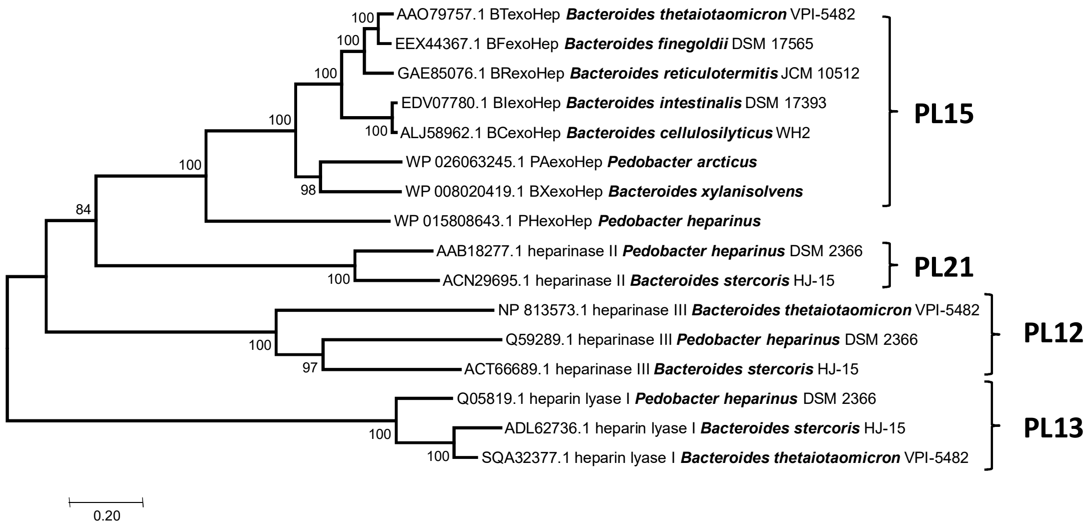

Supplementary Figure 1. Phylogenetic analysis of PL15_2 proteins. Phylogenetic analysis of exoHepases was carried out based on protein sequence alignments with reported Hepases in various Hepases families. The phylogenetic tree was constructed using MEGA version 7.0.26 software via the neighbor-joining algorithm and associated taxa clustered together in the bootstrap test of 1000 replicates.

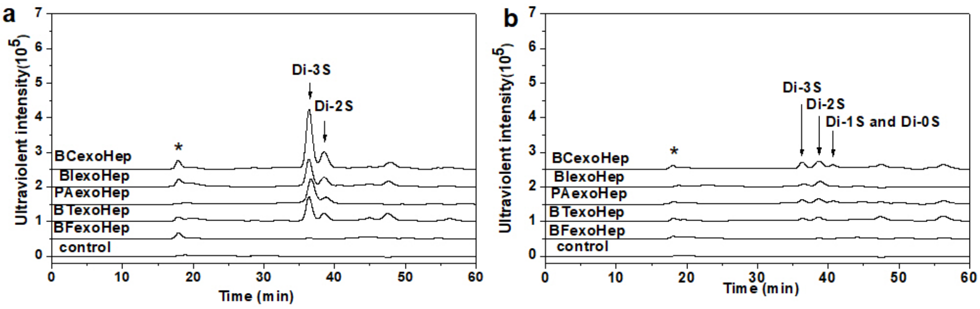

Supplementary Figure 2. Digestion of HP and HS by the novel Hepase in PL15_2 family. HP (**a**) or HS (**b**) polysaccharide (10 μg) was treated with each indicated enzyme for overnight. Each resultant was analyzed by SEC on a Superdex Peptide 10/300 GL column as described under "Methods". Di-3S, the trisulfated HP disaccharide; Di-2S, the disulfated HP disaccharide; Di-1S, the monosulfated HP disaccharide; Di-0S, the nonsulfated HP disaccharide; *, polysaccharides or proteins in void volume.

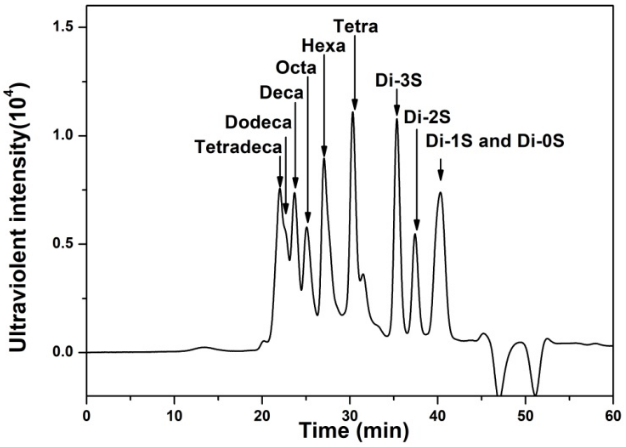

Supplementary Figure 3. The SEC separation of HP standard oligosaccharides. The size-defined HP oligosaccharides were analyzed by SEC on a Superdex Peptide 10/300 GL column as mentioned above. Tetradeca, the HP tetradecasaccharide; Dodeca, the HP dodecasaccharide; Deca, the HP decasaccharide; Octa, the HP octasaccharide; Hexa, the HP hexasaccharide; Tetra, the HP tetrasaccharide; Di-3S, the trisulfated HP disaccharide; Di-2S, the disulfated HP disaccharide; Di-1S, the monosulfated HP disaccharide; Di-0S, the nonsulfated HP disaccharide.

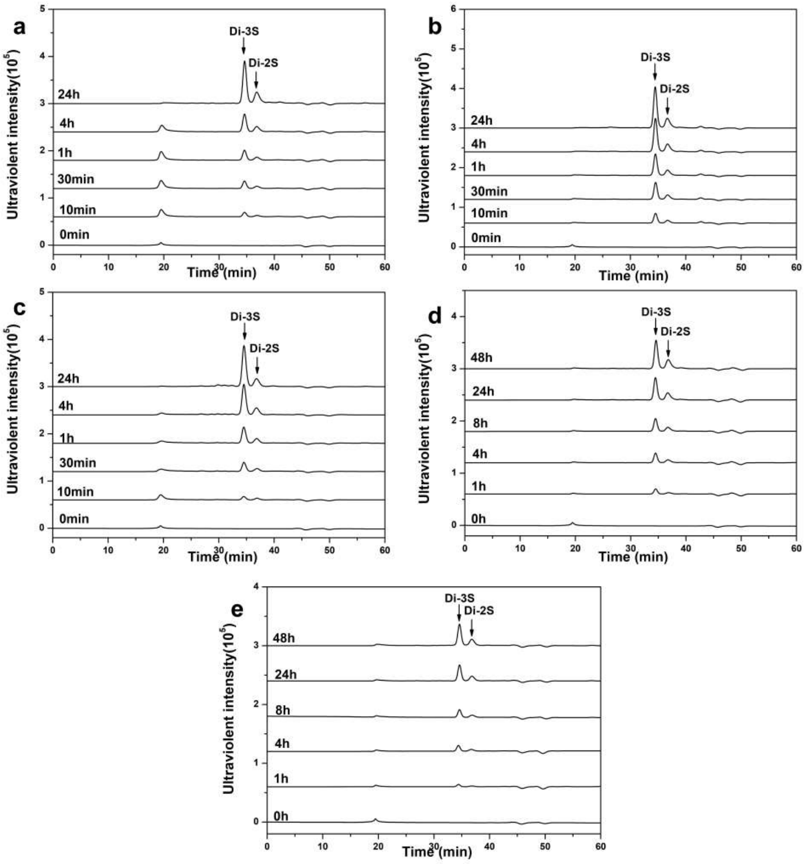

Supplementary Figure 4. Time-course assay of degradation of HP polysaccharides by PL15_2 family enzymes. HP polysaccharide (1 mg/ml) was treated with the enzyme (10 unit/ml) of BIexoHep (**a**), BCexoHep (**b**), PAexoHep (**c**), BTexoHep (**d**) or BFexoHep (**e**). Aliquots (20 μg) were taken at different time points for SEC analysis as described under “Methods”. Di-3S, the trisulfated HP disaccharide; Di-2S, the disulfated HP disaccharide. The elution positions of HP oligosaccharides on the SEC column were calibrated by using size-defined HP standard oligosaccharides as shown in Supplementary Fig. 3.

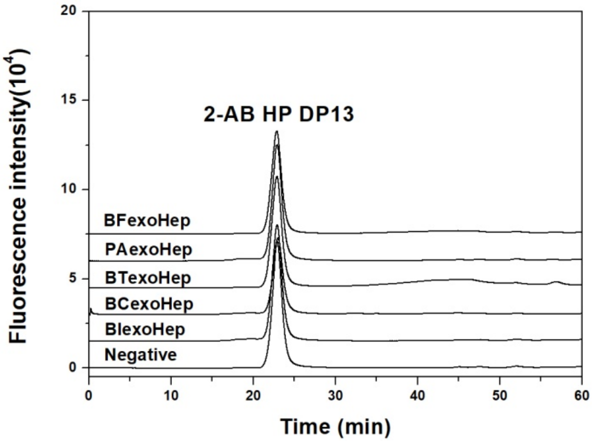

Supplementary Figure 5. Degradation of 2-AB labeled HP DP13 by PL15_2 family enzymes. One microgram of 2-AB labeled HP DP13 was treated by 5 mU proteins of BIexoHep, BCexoHep, PAexoHep, BTexoHep or BFexoHep, respectively. The resultant samples were analyzed as described under “Methods”.

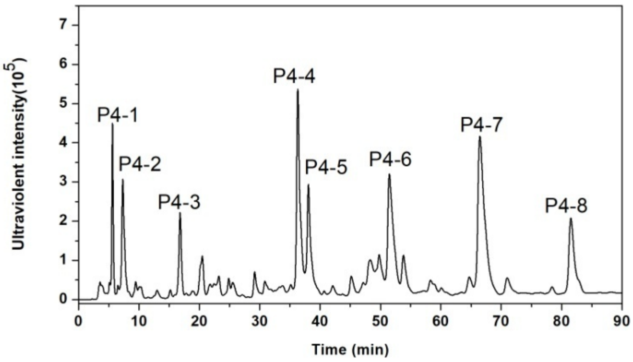

Supplementary Figure 6. Subfractionation of size-defined HP tetrasaccharides. The size-defined HP tetrasaccharide fraction prepared from the partial digestion of HP with Hepase II was further subfractionated by anion-exchange HPLC on a Pack Polyamine II column eluted with a NaH_2PO_4 gradient (0-1 M) and monitored with a UV detector at 232 nm.

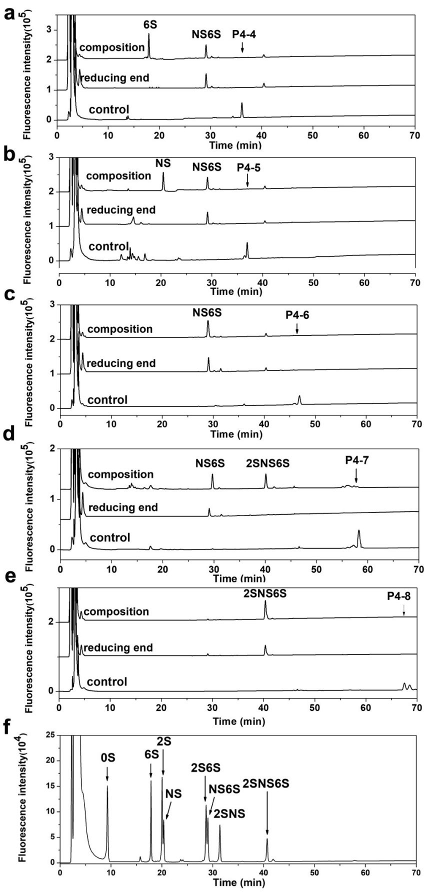

Supplementary Figure 7. Sequences of structure-defined HP tetrasaccharides. The disaccharides composition, reducing end disaccharide and untreated fraction (control) of tetrasaccharide subfractions P4-4 (**a**), P4-5 (**b**), P4-6 (**c**), P4-7 (**d**), or P4-8 (**e**) were analyzed by anion-exchange HPLC on a Pack Polyamine II column as described under “Methods”. The disaccharides were determined based on the elution positions of HP standard disaccharides on the anion-exchange column (**f**). 0S, ΔUA(1–4)GlcNAc; 6S, ΔUA(1–4)GlcNAc6S; 2S, ΔUA2S(1–4)GlcNAc; NS, ΔUA(1–4)GlcNS; 2S6S, ΔUA2S(1–4)GlcNAc6S; NS6S, ΔUA(1–4)GlcNS6S; 2SNS, ΔUA2S(1–4)GlcNS; and 2SNS6S, ΔUA2S(1–4)GlcNS6S.

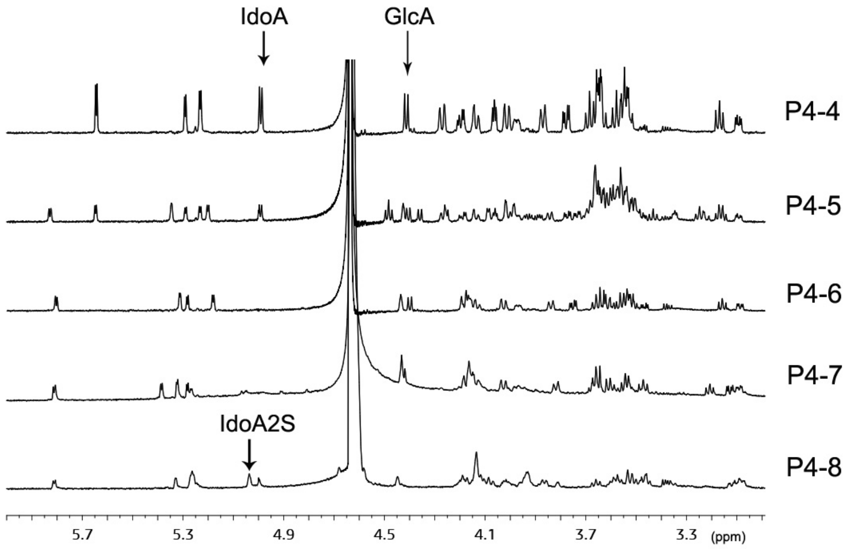

Supplementary Figure 8. ^{1}H NMR analyses of structure-defined HP tetrasaccharides. ^{1}H NMR (600 MHz) spectra of HP tetrasaccharide subfraction P4-4, P4-5, P4-6, P4-7 or P4-8 (400 μg) was obtained on a JNM-ECP600 instrument set at 600 MHz. The most relevant signals are pointed out as follows: the H-1 signal at 4.98 ppm indicates that the internal uronic acid type is IdoA, observed inP4-4 and P4-5; the H-1 signal at 5.04 ppm indicates that the internal uronic acid type is IdoA2S, observed in P4-8; and the H-1 signal at 4.42 ppm indicates that the internal uronic acid type is GlcA, observed in P4-4, P4-5, P4-6and P4-7.

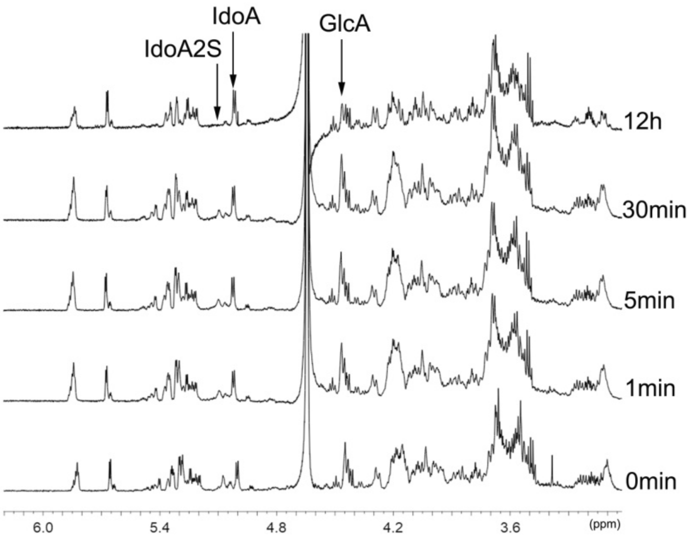

Supplementary Figure 9. ^{1}H NMR analyses of HP tetrasaccharides treated with BIexoHep. ^{1}H NMR (600 MHz) spectra of each 3 mg HP tetrasaccharides treated with 0.9 U BIexoHep for 0 min, 1 min, 5 min, 30 min and 12 h were obtained on a JNM-ECP600 instrument set at 600 MHz. The most relevant signals are pointed out as follows: the H-1 signal at 5.02 ppm indicates that the internal uronic acid type is IdoA; the H-1 signal at 5.10 ppm indicates that the internal uronic acid type is IdoA2S; and the H-1 signal at 4.47 ppm indicates that the internal uronic acid type is GlcA.

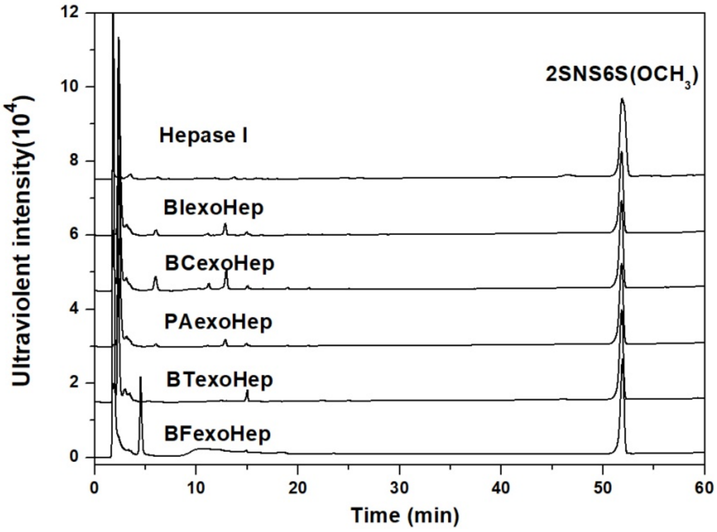

Supplementary Figure 10. Degradation of Arixtra by PL15_2 family enzymes. Arixtra (5 μg) was treated by BIexoHep, BCexoHep, PAexoHep, BTexoHep or BFexoHep, and the reactant was analyzed by anion-exchange HPLC as described under “Methods”. 2SNS6S(OCH_3), ΔUA2S(1–4)GlcNS6S(OCH_3).

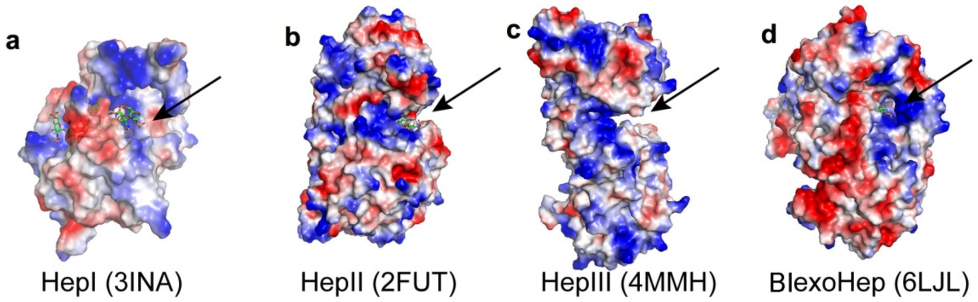

Supplementary Figure 11. Electrostatic and surface potential of various Hepases molecules. Electrostatic surface views of *Bacteroides thetaiotaomicron* Hepase I (**a**), *Pedobacter* Hepase II (**b**), *Pedobacter* Hepase III (**c**), *Bacteroides intestinalis* BIexoHep (**d**). The active clefts of the Hepases are denoted with arrows.

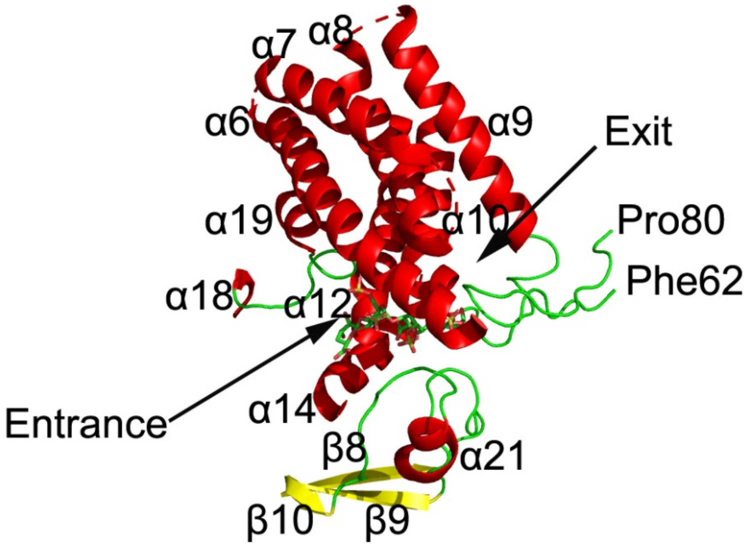

Supplementary Figure 12. Cartoon representation of the catalytic tunnel of BIexoHep. The α-helices and β-strands were colored in red and yellow, respectively. The entrance and exit were denoted with arrows.

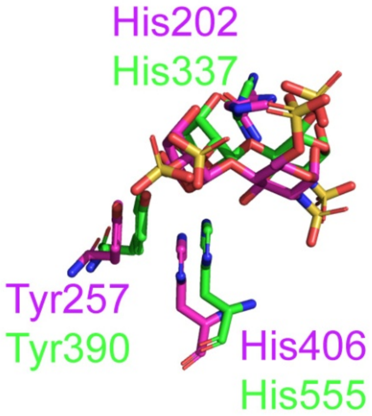

Supplementary Figure 13. Active sites overlap comparisons of BIexoHep with Hepase II. Overlap comparison of key residues and bound disaccharide products in active centers of *P. heprinus* DSM 2366 Hepase II and BIexoHep. The disaccharides and active residues were shown as sticks, in which the carbons of sugar rings is in yellow and the sidechain carbons of residues were colored pink and green, respectively.

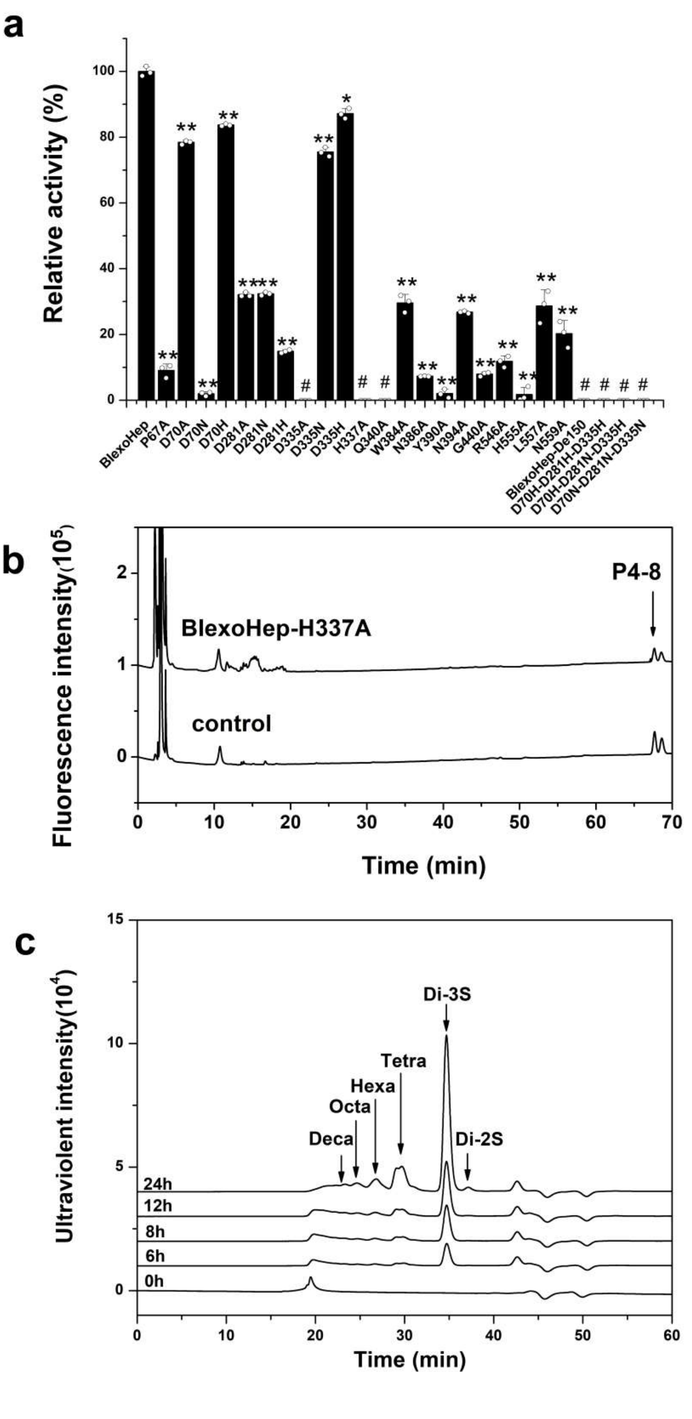

Supplementary Figure 14. Enzyme activities of BIexoHep mutants. (**a**) Residual enzyme activity of BIexoHep mutants. The relative activity of each mutant is shown as the

percentage of that (100%) of the WT-BIexoHep. BIexoHep-De150, a truncated mutant of BIexoHep without the N-terminal small β-sheet domain (residues 1-150). Error bars represent means of triplicates ± SD. *: P<0.001, **: P<0.0001, #: the activities of the mutants were lost or the activity were too low to accurately detected thus the significance were not compared with the control group. The p-values in all cases are compared with the corresponding control BIexoHep and provided in source data. Source data are provided in a Source Data file. (**b**) Degradation of P4-8 by BIexoHep-H337A and BIexoHep. The structure-defined tetrasaccharide P4-8 with high sulfation was treated with or without (control) the mutant BIexoHep-H337A and the resultants were labeled with 2-AB followed by anion-exchange HPLC as described under "Methods". (**c**) Time-course assay of degradation of HP polysaccharides by BIexoHep-D70H-D281N-D335H as described in the case of the WT-BIexoHep above. Deca, the HP decasaccharide; Octa, the HP octasaccharide; Hexa, the HP hexasaccharide; Tetra, the HP tetrasaccharide; Di-3S, the trisulfated HP disaccharide; Di-2S, the disulfated HP disaccharide

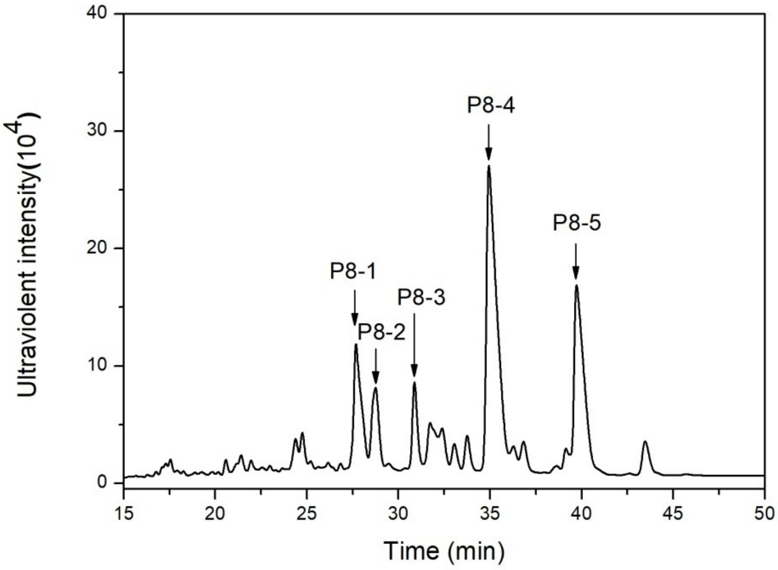

Supplementary Figure 15. Subfractionation of size-defined HP octasaccharides. The size-defined HP octasaccharide fraction prepared from the final products of HP with Hepase III was further subfractionated by anion-exchange HPLC on a Propack PA1 column eluted using a NaCl gradient (0.5-1.5 M) in 70 min by monitoring at 232 nm.

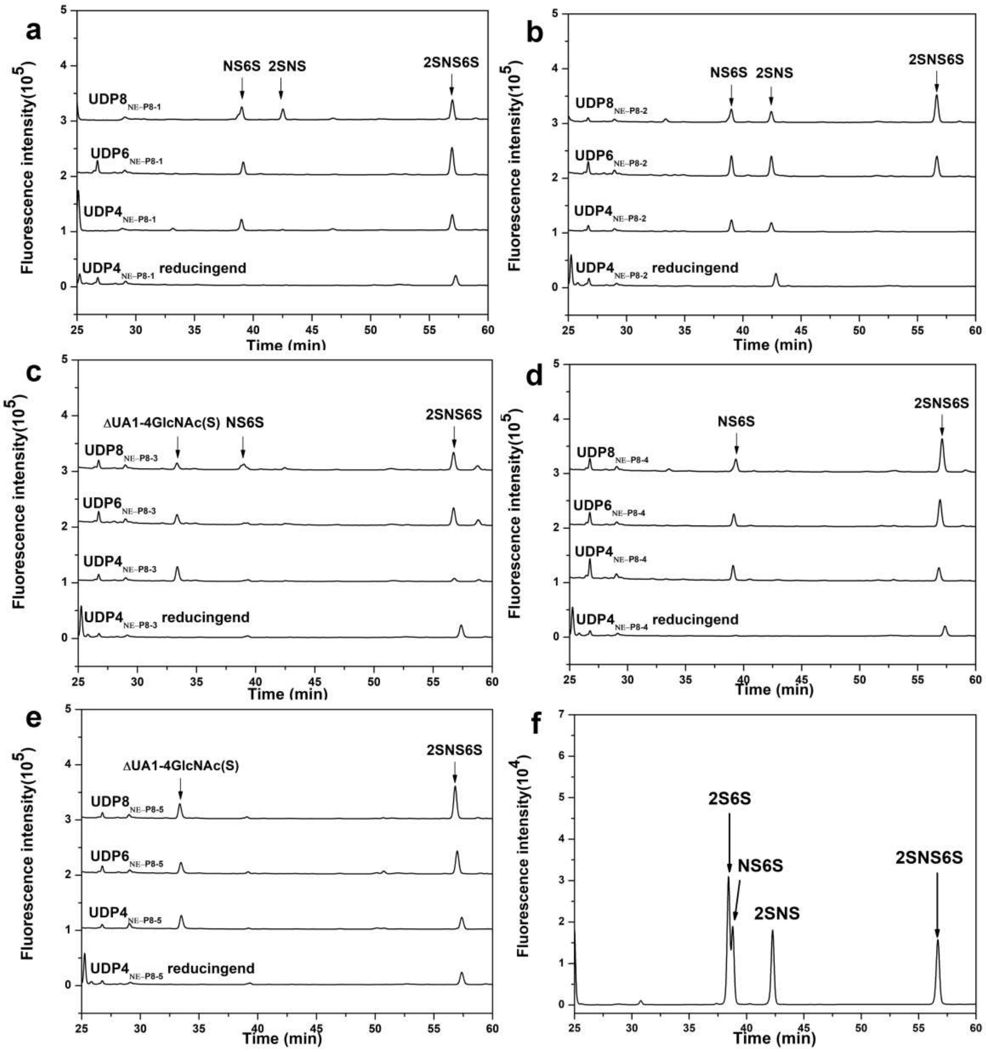

Supplementary Figure 16. Preliminary sequencing of Hepase III-resistant HP octasaccharides P8-1, P8-2, P8-3, P8-4 and P8-5. The preliminary sequences of P8-1 (**a**), P8-2 (**b**), P8-3 (**c**), P8-4 (**d**), and P8-5 (**e**) were determined by partial digestion with BIexoHep followed by disaccharide analysis of the resulted oligosaccharides through complete digestion combined with anion-exchange HPLC on a Pack Polyamine II column as described under “Methods”. The disaccharides were confirmed according to the standard HP disaccharides (**f**). ΔUA1-4GlcNAc(S): unknown monosulfated ΔUA(1–4)GlcNAc;

2S6S, ΔUA2S(1–4)GlcNAc6S; NS6S, ΔUA(1–4)GlcNS6S; 2SNS, ΔUA2S(1–4)GlcNS; 2SNS6S, ΔUA2S(1–4)GlcNS6S.

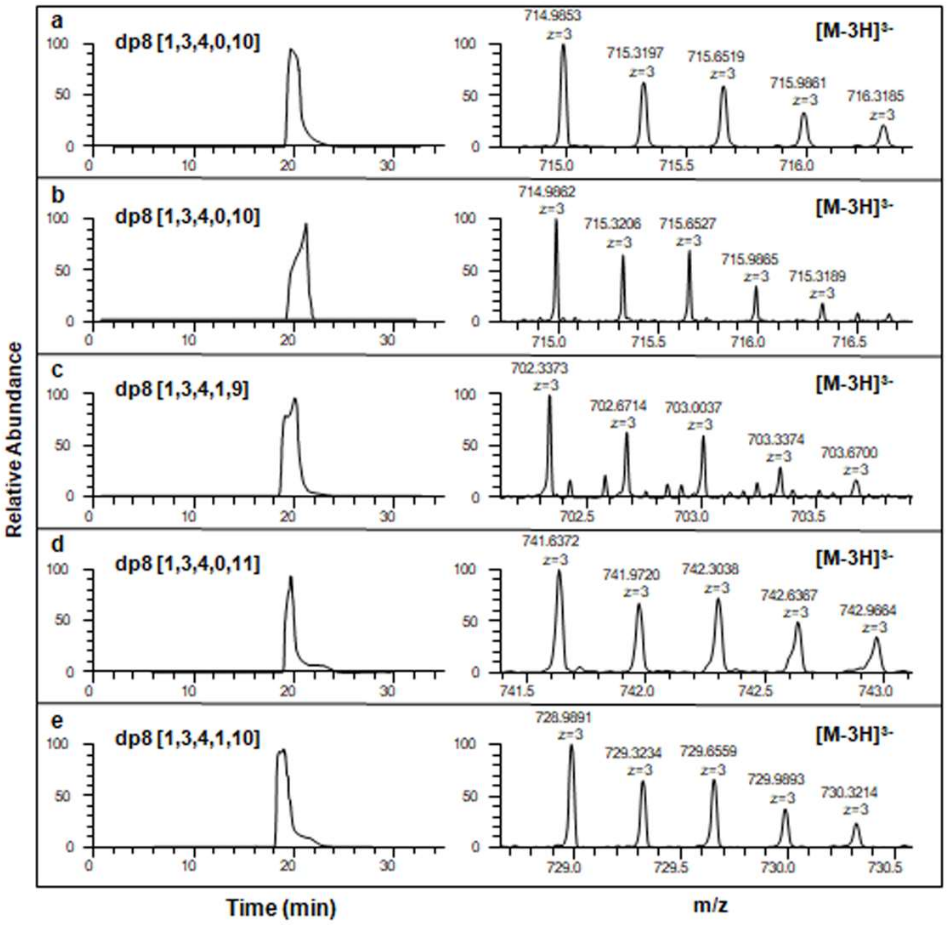

Supplementary Figure 17. Extracted ion chromatograms (EICs) and high resolution mass spectrograms of subfractionated HP octassacharides using HILIC-ESI-MS. (**a**) P8-1, (**b**) P8-2, (**c**) P8-3, (**d**) P8-4, (**e**) P8-5. The octasaccharides compositions were given as [ΔHexA, HexA, GlcN, Ac, SO_3].

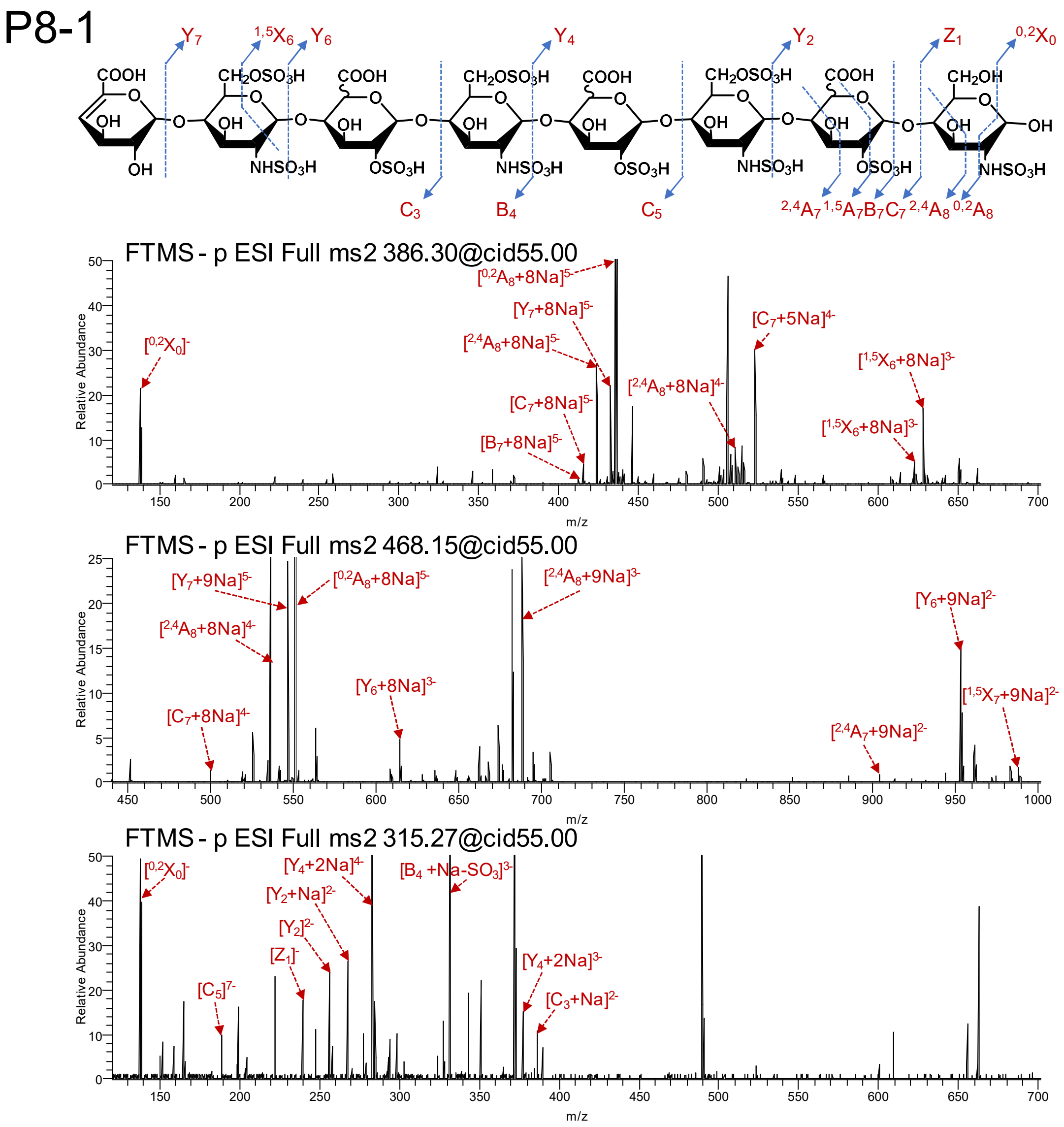

Supplementary Figure 18. The ESI-MS/MS spectra of P8-1.

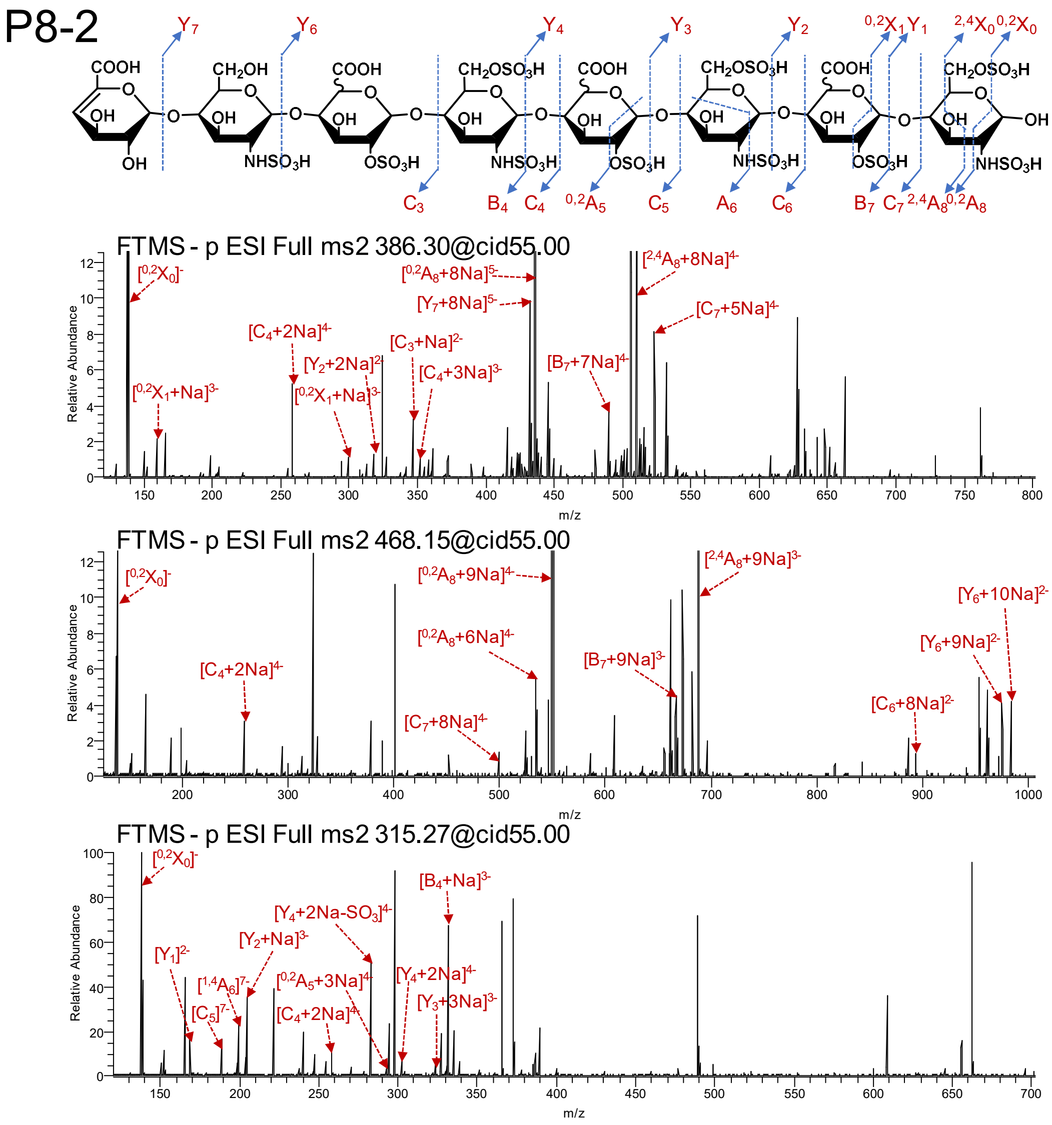

Supplementary Figure 19. The ESI-MS/MS spectra of P8-2.

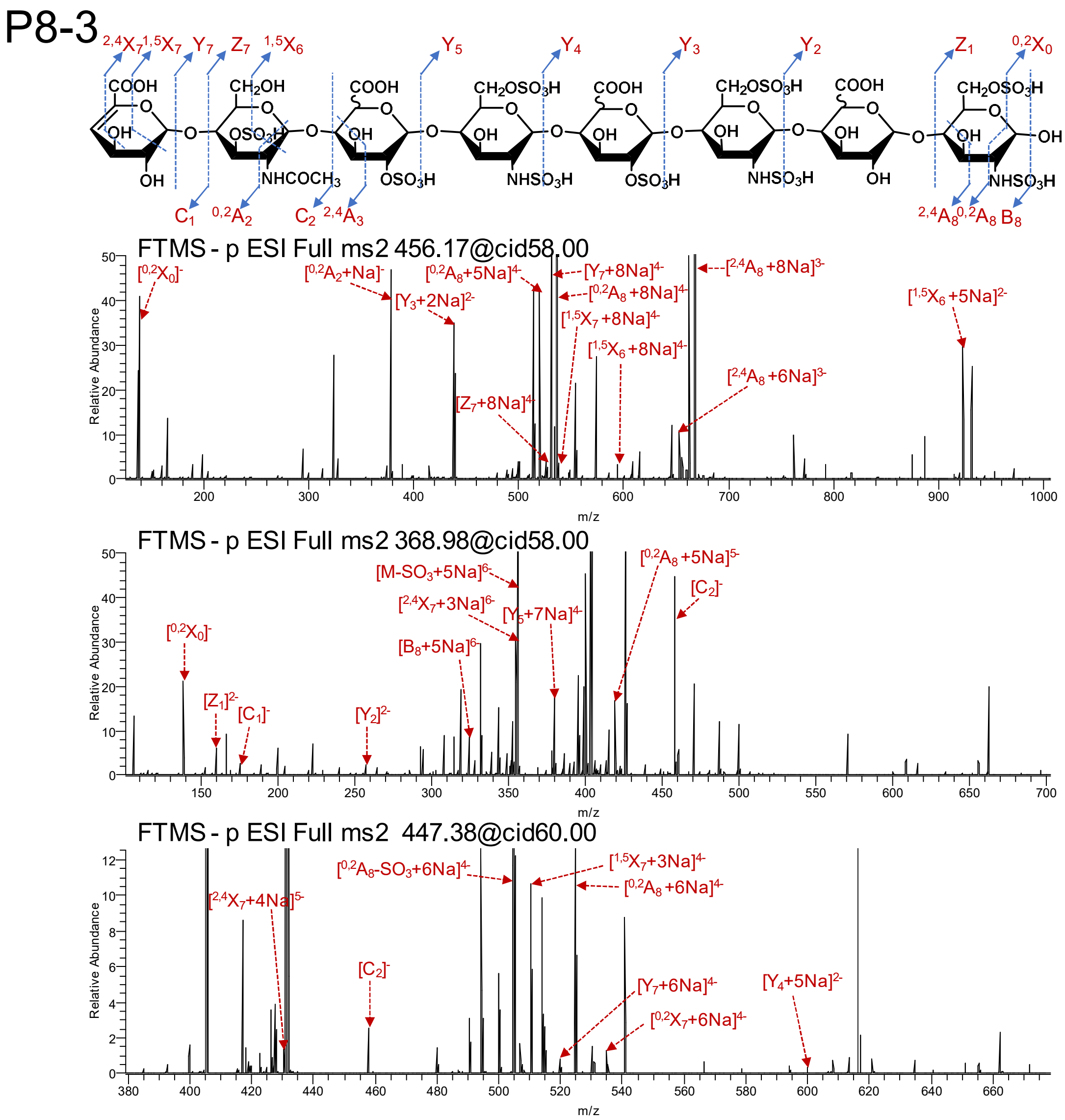

Supplementary Figure 20. The ESI-MS/MS spectra of P8-3.

P8-4

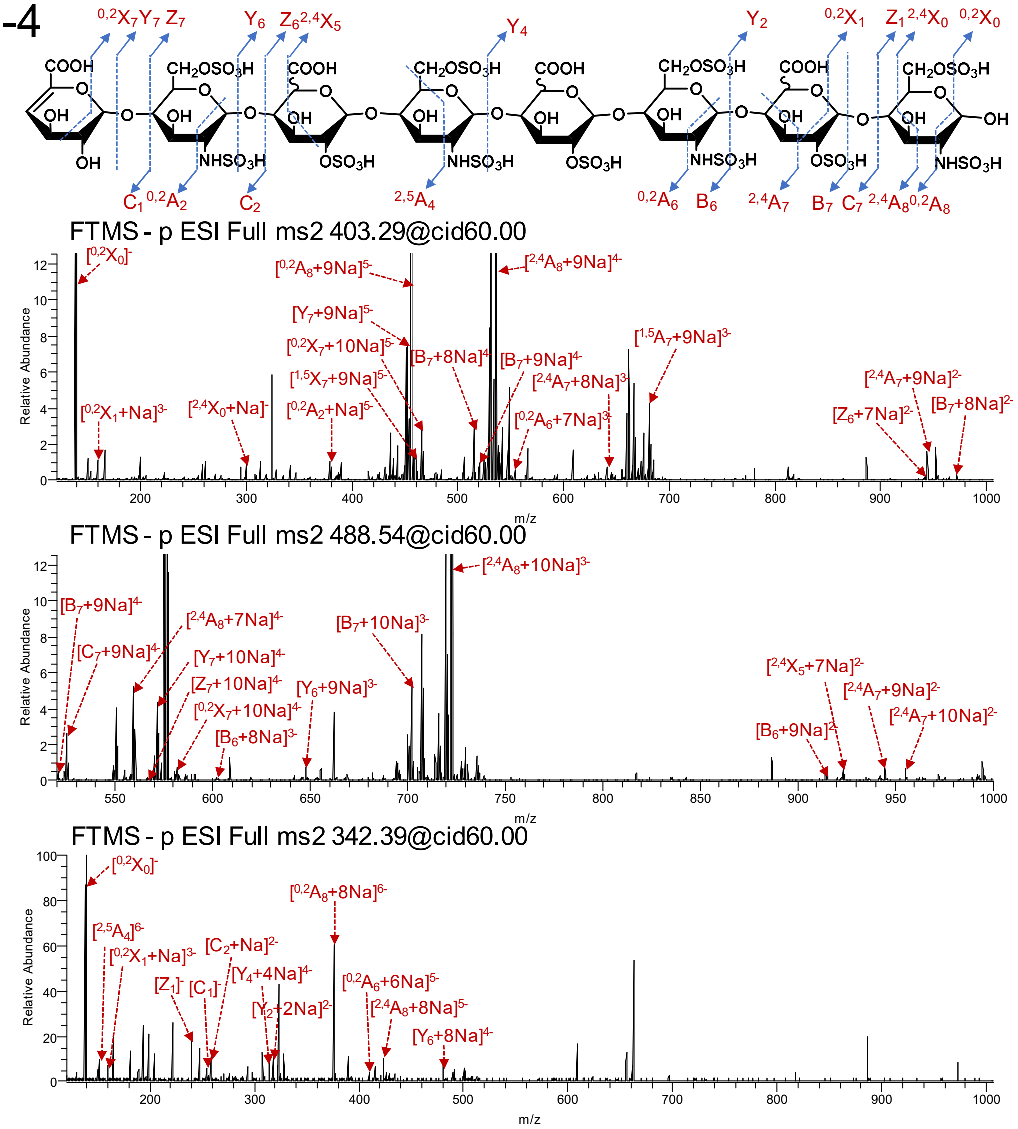

Supplementary Figure 21. The ESI-MS/MS spectra of P8-4.

P8-5

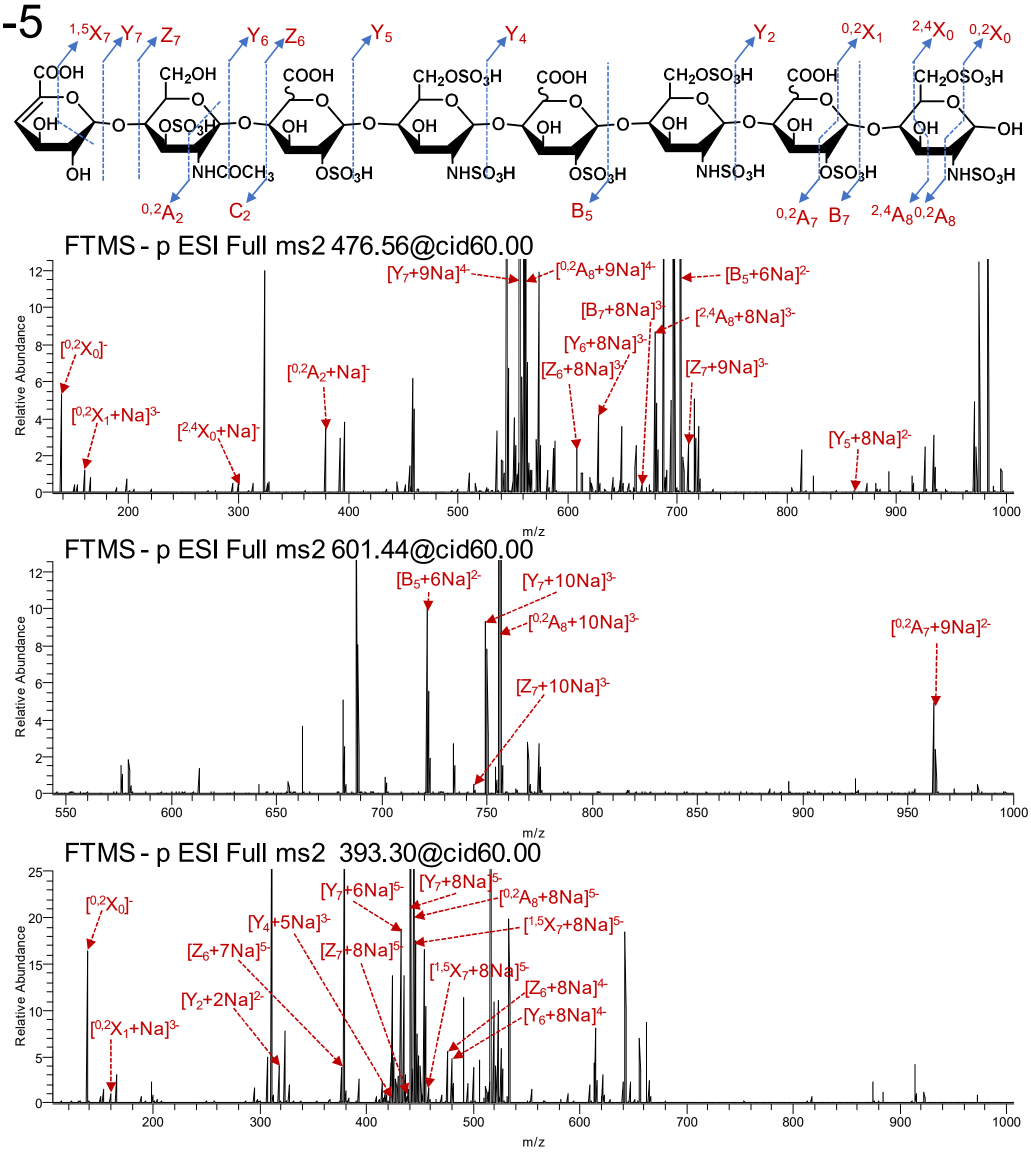

Supplementary Figure 22. The ESI-MS/MS spectra of P8-5.

Supplementary Tables

Supplementary Table 1. Sequences of the structure-defined HP tetrasaccharides.

	sequence
P4-4	ΔUA(1-4)GlcNAc6S(1-4)GlcA(1-4)GlcNS6S<br>ΔUA(1-4)GlcNAc6S(1-4)IdoA(1-4)GlcNS6S
P4-5	ΔUA(1-4)GlcNS(1-4)GlcA(1-4)GlcNS6S<br>ΔUA(1-4)GlcNS(1-4)IdoA(1-4)GlcNS6S
P4-6	ΔUA(1-4)GlcNS6S(1-4)GlcA(1-4)GlcNS6S
P4-7	ΔUA2S(1-4)GlcNS6S(1-4)GlcA(1-4)GlcNS6S
P4-8	ΔUA2S(1-4)GlcNS6S(1-4)IdoA2S(1-4)GlcNS6S

Supplementary Table 2. The conversion rates of the tetrasaccharides.

	P4-4	P4-5	P4-6	P4-7	P4-8
Conversion rate (%)	15.90	9.37	34.68	100	100

Supplementary Table 3. The enzyme activities of BIexoHep toward the HP tetrasaccharides.

	P4-4	P4-5	P4-6	P4-7	P4-8
Enzyme activities (U/mg)	<1	3.89	24.22	49.12	79.49

Supplementary Table 4. The molar ratio of IdoA2S, IdoA and GlcA contents in ^{1}H NMR (600 MHz) spectra of HP tetrasaccharides treated with BIexoHep for various time.

Reaction time	Molar ratio of each content		
	IdoA2S	IdoA	GlcA
0 min	1.00	1.74	2.34
1 min	1.00	1.90	3.20
5 min	1.00	2.00	3.43
30 min	1.00	2.48	4.52
12 h	-	1.00	0.50

Supplementary Table 5. The theoretically molar ratio of the different disaccharides in the parental octasaccharides and their nonreducing end oligosaccharides released by the partial digestion with BIexoHep.

		Molar ratio of the disaccharides			
		ΔUA1-4GlcNAc(S)	NS6S	2SNS	2SNS6S
P8-1	Octa	-	1	1	2
	nHexa	-	1	-	2
	nTetra	-	1	-	1
P8-2	Octa	-	1	1	2
	nHexa	-	1	1	1
	nTetra	-	1	1	-
P8-3	Octa	1	1	-	2
	nHexa	1	-	-	2
	nTetra	1	-	-	1
P8-4	Octa	-	1	-	3
	nHexa	-	1	-	2
	nTetra	-	1	-	1
P8-5	Octa	1	-	-	3
	nHexa	1	-	-	2
	nTetra	1	-	-	1

Octa, octasaccharide; nHexa and nTetra, the nonreducing end hexa- and tetrasaccharides of each parental octasaccharide; ΔUA1-4GlcNAc(S): unknown monosulfated ΔUA(1–4)GlcNAc; NS6S, ΔUA(1–4)GlcNS6S; 2SNS, ΔUA2S(1–4)GlcNS; 2SNS6S, ΔUA2S(1–4)GlcNS6S.

Supplementary Table 6. Preliminary sequences of the Hepase III-resistant HP octasaccharides.

	Sequences of the octasaccharides
P8-1	ΔUA1-4GlcNS6S1-4HexA2S1-4GlcNS6S-4HexA2S1-4GlcNS6S1-4HexA2S1-4GlcNS
P8-2	ΔUA1-4GlcNS6S1-4HexA2S1-4GlcNS-4HexA2S1-4GlcNS6S1-4HexA2S1-4GlcNS6S
P8-3	ΔUA1-4GlcNAc(S)1-4HexA2S1-4GlcNS6S-4HexA2S1-4GlcNS6S1-4HexA1-4GlcNS6S
P8-4	ΔUA1-4GlcNS6S1-4HexA2S1-4GlcNS6S1-4HexA2S1-4GlcNS6S1-4HexA2S1-4GlcNS6S
P8-5	ΔUA1-4GlcNAc(S)1-4HexA2S1-4GlcNS6S1-4HexA2S1-4GlcNS6S1-4HexA2S1-4GlcNS6S

Supplementary Table 7. Major components of the Hepase III-resistant HP octasaccharides identified using HILIC-ESI-MS.

Component	m/z	Charge	MW $_{experimental}$ (Da)	Structure	Mw $_{theoretical}$ (Da)	Mass accuracy (ppm)
P8-1	714.9853	3	2147.9794	dp8 [1,3,4,0,10]	2147.9717	3.58
P8-2	714.9862	3	2147.9821	dp8 [1,3,4,0,10]	2147.9717	4.84
P8-3	702.3373	3	2110.0378	dp8 [1,3,4,1,9]	2110.0255	4.71
P8-4	741.6372	3	2227.9351	dp8 [1,3,4,0,11]	2227.9285	2.95
P8-5	728.9891	3	2189.9908	dp8 [1,3,4,1,10]	2189.9823	3.90

Supplementary Table 8. Primer pairs of mutation.

Muntants		Primers
BlexoHep-P67A	F	TTTAGGTgcgGTGTTGGATGGAGTTCCGGGAC
	R	CCAACACcgcACCTAAATGAGGGAATTTGTCCG
BlexoHep-D70A	F	GGTGTTGgcgGGAGTTCCGGGACAAGTAGATG
	R	GAACTCCcgcCAACACCGGACCTAAATGAGGG
BlexoHep-D70NA	F	GGTGTTGaacGGAGTTCCGGGACAAGTAGATG
	R	GAACTCCgttCAACACCGGACCTAAATGAGGG
BlexoHep-D281A	F	TGCAGGCgcgTTTAACCTGTCCACCCTATTATCGA
	R	GGTTAAAcgcGCCTGCAAAGTATTTGCTTTTTT
BlexoHep-D281N	F	TGCAGGCaacTTTAACCTGTCCACCCTATTATCGA
	R	GGTTAAAgttGCCTGCAAAGTATTTGCTTTTTT
BlexoHep-D335A	F	TATTGCAgcgAACCATGTGTGGCAGATGACTTT
	R	CATGGTTcgcTGCAATACGGTTCTCCAGATGG
BlexoHep-D335N	F	CCGTATTGCAaacAACCATGTGTGGCAGATGACTTT
	R	GGTTgttTGCAATACGGTTCTCCAGATGGTTC
BlexoHep-H337A	F	ATTGCAGACAACgcgGTGTGGCAGATGACTTTCCGTA
	R	ACcgcGTTGTCTGCAATACGGTTCTCCAGATG
BlexoHep-Y390A	F	ATGCCgcgTTCCATGTAAACATACGTACCTTGATAG
	R	TACATGGAAcgcGGCATCCCCATTGTGCCAGC
BlexoHep-H555A	F	ATGGTTCCACATCCgcgGCCTTGGCCAATCAAAACG
	R	cgcGGATGTGGAACCATAAGGACTGCTTCGGA
BlexoHep-Q340A	F	TGTGTGGgcgATGACTTTCCGTATCCTGACGATG
	R	AAGTCATcgcCCACACATGGTTGTCTGCAATAC
BlexoHep-W384A	F	ATGGTGGCgcgCACAATGGGGATGCCTATTTCC
	R	ATTGTGcgcGCCACCATCCTTATGTAGGCCCG
BlexoHep-N386A	F	gcgGGGGATGCCTATTTCCATGTAAACATACG
	R	AAATAGGCATCCCCcgcGTGCCAGCCACCATCCTTATG
BlexoHep-N394A	F	CCATGTAgcgATACGTACCTTGATAGAAGTGCCG
	R	TACGTATcgcTACATGGAAATAGGCATCCCCA
BlexoHep-G440A	F	GGCATgcgAACTCACATGAAGGACAACGCAGT
	R	ATGTGAGTTcgcATGCCCTCCGGATTTGGAGA

BlexoHep-R546A	F	TTCTTTCgcgAGCAGTCCTTATGGTTCCACATC
	R	GACTGCTcgcGAAAGAAAGCATGGCATTGTTG
BlexoHep-L557A	F	ATGCCgcgGCCAATCAAAACGCTTTCAACACC
	R	TTGATTGGCcgcGGCATGGGATGTGGAACCAT
BlexoHep-N559A	F	CgcgCAAAACGCTTTCAACACCTTCTTTGGGG
	R	TGAAAGCGTTTTGcgcGGCCAAGGCATGGGATGT

Supplementary Table 9. Data collection and refinement statistics.

Parameters	BlexoHep-HP di	SeMet-BlexoHep-HP di	Y390A/H555A-HP tetra
Data collection			
Space group	P 2_1 2_1 2_1	P 2_1 2_1 2_1	P 2_1 2_1 2_1
Wavelength(Å)	0.9792	0.9792	0.9792
Unit cell parameters	a=59.475 Å	a=59.235 Å	a=59.459 Å
	b=108.333 Å	b=107.846 Å	b=107.851 Å
	c=128.685 Å	c=128.963 Å	c=129.434 Å
	α=90°	α=90°	α=90°
	β=90°	β=90°	β=90°
	γ=90°	γ=90°	γ=90°
Resolution range (Å)	34.77-1.978 (2.049-1.978)	25.09-1.699 (1.759-1.699)	82.86-1.734 (1.795-1.734)
Unique reflections	58145 (5338)	91225 (8773)	72171 (7702)
Redundancy	6.4 (6.2)	12.7 (12.7)	10.5 (5.4)
Completeness (%)	98.84 (91.72)	99.45 (96.99)	82.90 (89.50)
R_{merge}[a]	0.101 (0.309)	0.101 (0.487)	0.059(0.329)
Mean $I/\sigma(I)$	15.17 (5.01)	30.55 (7.66)	22.23 (3.02)
Refinement statistics			
R_{work}[b]	0.149 (0.157)		0.164 (0.209)
R_{free}	0.184 (0.213)		0.203 (0.264)
Average B-factor (Å^2)	22.7		25.3
Protein	21.8		24.2
Water	29.0		30.8
Ligand	26.8		64.9
Ion	23.0		22.3
r.m.s.d. from ideal geometry			
r.m.s.d. length (Å)	0.022		0.007
r.m.s.d. angles (°)	1.22		0.88
Ramachandran Plot (%)			
Favored	97.5		97.0
Allowed	2.5		2.9
Outliers	0		0.1

[a]$R_{merge}=\Sigma_{hkl}\Sigma_i|I_i(hkl) - \langle I(hkl)\rangle|/\Sigma_{hkl}\Sigma_i \langle I_i(hkl)\rangle$

[b]$R_{work}=\sum_{hkl}||F_{obs}| - |F_{calc}||/\sum_{hkl} (F_{obs})$

Supplementary Reference

1. Myette, J. R. et al. The heparin/heparan sulfate 2-*O*-sulfatase from *Flavobacterium heparinum*. Molecular cloning, recombinant expression, and biochemical characterization. *J. Biol. Chem*. **278,** 12157-12166 (2003).
2. Myette, J. R. et al. Molecular cloning of the heparin/heparan sulfate delta 4,5 unsaturated glycuronidase from *Flavobacterium heparinum*, its recombinant expression in *Escherichiacoli*, and biochemical determination of its unique substrate specificity. *Biochemistry*. **41,** 7424-7434 (2002).
3. Masuko, S. et al. Ozonolysis of the double bond of the unsaturated uronate residue in low-molecular-weight heparin and K5 heparosan. *Carbohydr. Res*. **346,** 1962-6 (2011).
4. Zhang, X. et al. Chemoenzymatic synthesis of heparan sulfate and heparin oligosaccharides and NMR analysis: paving the way to a diverse library for glycobiologists. *CHEM. SCI*. **8,** 7932-7940 (2017).
