# Supplementary material for: Discovery of exolytic heparinases and their catalytic mechanism and potential application
Source: Nat Commun. 2021 Feb 24;12:1263. doi: 10.1038/s41467-021-21441-8 (PMC7904915; doi:10.1038/s41467-021-21441-8)
Supplement: Supplementary file 3 — Reporting Summary [file 41467_2021_21441_MOESM3_ESM.pdf]

nature research

Corresponding author(s): Fuchuan Li

Last updated by author(s): Jan 11, 2021

Reporting Summary

Nature Research wishes to improve the reproducibility of the work that we publish. This form provides structure for consistency and transparency in reporting. For further information on Nature Research policies, see our Editorial Policies and the Editorial Policy Checklist.

Statistics

For all statistical analyses, confirm that the following items are present in the figure legend, table legend, main text, or Methods section.

n/a	Confirmed	
☐	☒	The exact sample size (n) for each experimental group/condition, given as a discrete number and unit of measurement
☐	☒	A statement on whether measurements were taken from distinct samples or whether the same sample was measured repeatedly
☐	☒	The statistical test(s) used AND whether they are one- or two-sided<br>*Only common tests should be described solely by name; describe more complex techniques in the Methods section.*
☒	☐	A description of all covariates tested
☒	☐	A description of any assumptions or corrections, such as tests of normality and adjustment for multiple comparisons
☐	☒	A full description of the statistical parameters including central tendency (e.g. means) or other basic estimates (e.g. regression coefficient) AND variation (e.g. standard deviation) or associated estimates of uncertainty (e.g. confidence intervals)
☒	☐	For null hypothesis testing, the test statistic (e.g. F, t, r) with confidence intervals, effect sizes, degrees of freedom and P value noted<br>*Give P values as exact values whenever suitable.*
☒	☐	For Bayesian analysis, information on the choice of priors and Markov chain Monte Carlo settings
☒	☐	For hierarchical and complex designs, identification of the appropriate level for tests and full reporting of outcomes
☒	☐	Estimates of effect sizes (e.g. Cohen's d, Pearson's r), indicating how they were calculated

Our web collection on statistics for biologists contains articles on many of the points above.

Software and code

Policy information about availability of computer code

Data collection	LC solution version 1.25-HPLC data acquisition ;<br>BL17U1 beam line-crystal data acquisition;<br>Agilent 600MHz-NMR data acquistion;<br>Thermo Xcalibur 2.1.0-MS data acquistion;
Data analysis	Excel 2007 (Microsoft), Origin 8.5 (OriginLab)-figure preparation and statistical test calculation;<br>MestReNova (9.0.1)-NMR data analysis;<br>MEGA 7.0.26-Phylogenetic analysis;<br>Phaser, AutoSol, AutoBuild and Refine programs of Phenix (1.17.1-3660) and Wincoot 0.8.1- structure construction;<br>Pymol (TM) 2.2.0-structure analysis.

For manuscripts utilizing custom algorithms or software that are central to the research but not yet described in published literature, software must be made available to editors and reviewers. We strongly encourage code deposition in a community repository (e.g. GitHub). See the Nature Research guidelines for submitting code & software for further information.

Data

Policy information about availability of data

All manuscripts must include a data availability statement. This statement should provide the following information, where applicable:

- Accession codes, unique identifiers, or web links for publicly available datasets
- A list of figures that have associated raw data
- A description of any restrictions on data availability

All data supporting the findings of this study are available within the paper (and its Supplementary Information files). All relevant data generated during this study or

analyzed in this published article (and its Supplementary Information files) are available from the corresponding author on reasonable request. Source data are provided with this paper. The atomic coordinates and structure factors of the structures in this study have been deposited in the Protein Data Bank, www.pdb.org (PDB ID codes 6LJA (http://doi.org/10.2210/pdb6LJA/pdb) and 6LJL (http://doi.org/10.2210/pdb6LJL/pdb)). The sequences of BIexoHep (GenBank: EDV07780.1), BTexoHep (GenBank: AAO79757.1), BCexoHep (GenBank: ALJ58962.1), PAexoHep (GenBank: WP_026063245.1) and BFexoHep (GenBank: EEX44367.1) have already exist in NCBI database. The raw MS data of P8-1, P8-2, P8-3, P8-4, and P8-5 were uploaded onto figshare and can be accessed by the link http://doi.org/10.6084/m9.figshare.13413584.

Field-specific reporting

Please select the one below that is the best fit for your research. If you are not sure, read the appropriate sections before making your selection.

- [x] Life sciences
- [ ] Behavioural & social sciences
- [ ] Ecological, evolutionary & environmental sciences

For a reference copy of the document with all sections, see nature.com/documents/nr-reporting-summary-flat.pdf

Life sciences study design

All studies must disclose on these points even when the disclosure is negative.

Sample size	Several independent experiments were conducted, analyzed, and summarized in advance. Based on the resultant data repetition and deviation across measurements, and combining the similar studies in this field the suitable sample sizes were chosen.
Data exclusions	In general, no data was excluded.
Replication	Experiments were performed in at least three independent biological replicates.<br>All attempts at replication were successful and gave similar results.
Randomization	Randomization was not used and relevant to this study, since it did not involved an allocation of an intervention or a trial with human or animal participants.
Blinding	No blinding was used or necessary, as all the biochemical, chemical and biological experiments generated quantitative data.

Reporting for specific materials, systems and methods

We require information from authors about some types of materials, experimental systems and methods used in many studies. Here, indicate whether each material, system or method listed is relevant to your study. If you are not sure if a list item applies to your research, read the appropriate section before selecting a response.

Materials & experimental systems

n/a	Involved in the study	
x		Antibodies
x		Eukaryotic cell lines
x		Palaeontology and archaeology
x		Animals and other organisms
x		Human research participants
x		Clinical data
x		Dual use research of concern

Methods

n/a	Involved in the study	
x		ChIP-seq
x		Flow cytometry
x		MRI-based neuroimaging
